# scTransSort: Transformers for Intelligent Annotation of Cell Types by Gene Embeddings

**DOI:** 10.3390/biom13040611

**Published:** 2023-03-28

**Authors:** Linfang Jiao, Gan Wang, Huanhuan Dai, Xue Li, Shuang Wang, Tao Song

**Affiliations:** 1 College of Computer Science and Technology, China University of Petroleum, Qingdao 266580, China; 2 Department of Artificial Intelligence, Faculty of Computer Science, Campus de Montegancedo, Polytechnical University of Madrid, Boadilla del Monte, 28660 Madrid, Spain

**Keywords:** scRNA-seq, cell type, classification, annotation, identity, transformer

## Abstract

Single-cell transcriptomics is rapidly advancing our understanding of the composition of complex tissues and biological cells, and single-cell RNA sequencing (scRNA-seq) holds great potential for identifying and characterizing the cell composition of complex tissues. Cell type identification by analyzing scRNA-seq data is mostly limited by time-consuming and irreproducible manual annotation. As scRNA-seq technology scales to thousands of cells per experiment, the exponential increase in the number of cell samples makes manual annotation more difficult. On the other hand, the sparsity of gene transcriptome data remains a major challenge. This paper applied the idea of the transformer to single-cell classification tasks based on scRNA-seq data. We propose scTransSort, a cell-type annotation method pretrained with single-cell transcriptomics data. The scTransSort incorporates a method of representing genes as gene expression embedding blocks to reduce the sparsity of data used for cell type identification and reduce the computational complexity. The feature of scTransSort is that its implementation of intelligent information extraction for unordered data, automatically extracting valid features of cell types without the need for manually labeled features and additional references. In experiments on cells from 35 human and 26 mouse tissues, scTransSort successfully elucidated its high accuracy and high performance for cell type identification, and demonstrated its own high robustness and generalization ability.

## 1. Introduction

The rapid development of scRNA-seq technology and high-resolution transcriptome data have deepened our understanding of cellular phenotypic heterogeneity and complex tissue composition [1,2,3]. scRNA-seq has emerged as a powerful method to quantify the transcriptome of individual cells, and cell types can be determined from the entire transcriptome of thousands of individual cells [4,5]. However, data from scRNA-seq experiments are often noisy, high-dimensional, and highly sparse, and efficient computational analysis methods are urgently needed [6].

Cell type classification in the dataset is one of the most important steps in single-cell data analysis, and this step focuses on cell type identification using scRNA-seq data. Current strategies fall into two main types, one in which cells are clustered into clusters based on the similarity of gene expression profiles under supervision, and the annotation of cell clusters is achieved by manually assigning tags to each cluster. Including Scanpy [7], Seurat [8], SIMLR [9], SC3 [10], etc. belong to this type of method. This method proved to be valuable in identifying new cell populations [11,12,13,14,15,16]. However, this annotation step is tedious and time-consuming, because it involves manual examination of cluster-specific marker genes and requires a priori knowledge of known cellular markers. In addition, manual annotations that are not usually based on standardized cell-labeling ontologies are not reproducible across experiments [17]. As technology has evolved, a scRNA-seq experiment provides information about all genes, which is useful for revealing new biology, but making comparisons of high-dimensional data is very difficult, and there is a great deal of redundant or confounding information in high-dimensional distributions, and when comparing cells in a high-dimensional gene expression space, the distance between cells becomes more homogeneous, making it difficult to distinguish differences in populations. These drawbacks limit the ability of unsupervised methods to be annotated quickly and reproducibly [18,19]. Therefore, more and more automatic cell type recognition classification methods are starting to be used to overcome these challenges in scRNA-seq experiments [20,21], the second strategy: the semi-supervised and unsupervised cases. By comparing similarities between individual cells and a reference database of bulk or single-cell RNA-seq profiles, this type of classification method determines potential cell identity. Although all classification methods based on scRNA-seq data share a common goal of accurately annotating cells, these semi-supervised or unsupervised methods differ in the combination of underlying algorithms and a priori knowledge (e.g., lists of cell type marker genes) [22]. For example, SingleR, proposed by Aran D et al. [23], identifies cell types by calculating correlations based on the gene expression profile of each cell in the reference dataset. According to De Kanter J.K. et al. [24], CHETAH serves as a classification tree using scRNA-seq reference data. In the scMap proposed by Kiselev V.Y. et al. [25], cells are classified by their similarity to reference cell types based on various correlation measures. Fisher’s linear discriminant analysis-like framework is used in the scID suggested by Boufea K. et al. [26] to identify transcriptionally important cell types. The scPred method proposed by Alquicira-Hernandez J. et al. [27] combines identifying characteristics with solving the variance structure of the gene expression matrix. The ACTINN proposed by Ma F. et al. [28] uses a simple DNN, but it has the disadvantages of limited generalization ability and easy overfitting of the model. CellAssign introduced by Zhang A.W. et al. [29] is a probabilistic model using a hierarchical statistical framework, but it is suitable for the presence of well-understood marker genes and has limited performance for poorly characterized cell-type models. Garnett, presented by Pliner H.A. et al. [30], is a cell classification tool based on an interpretable hierarchical marker language for cell type-specific genes. The SCINA suggested by Zhang Z. et al. [31] is a semi-supervised model using the expectation-maximization (EM) algorithm, but it is highly dependent on the feature genes, and a more stable performance can be obtained only by including more feature genes. Tan Y. et al. [32] proposed singleCellNet to construct a cell classification model using a random forest classifier. Using a weighted graph neural network deep learning model for cell type detection, Shao X. et al. [33] developed scDeepSort. However, the accuracy in identifying cell types using the above methods is still limited, and the task of performing cell type identification of single-cell transcriptome data remains a challenge, probably because these methods may ignore the a priori knowledge of the transcriptome and the possible structure of the data [34,35]. In addition, these methods are sensitive to data sparsity, which means that the performance of the model may be significantly degraded when dealing with transcriptomic data with severe data sparsity.

To solve the above problems, we propose a deep neural network model scTransSort based on transformer pretraining, which intelligently extracts features from scRNA-seq data and predicts cell types through a self-attentive mechanism. The transformer model based on the self-attention architecture has been a great success not only in the field of natural language processing but also in the field of image recognition. However, according to the current research, there are almost no tools that apply the transformer model for cell type identification in processing scRNA-seq. The transformer has high computational efficiency and scalability to train models of large sizes with more than 100 B parameters (Brown et al., 2020 [36]; Lepikhin et al., 2020 [37]), and there is no performance saturation as the dataset grows [38], which makes us very expectant about the performance of the transformer on high-dimensional scRNA-seq data. In this paper, the transformer model was migrated to a single-cell classification task based on scRNA-seq data, and the effect of the model was tested on independent datasets of cells from 35 human tissues and 26 mouse tissues, respectively, including bladder, brain, lungs, muscles, pancreas, ovaries, spleen, etc. Finally, the performance is compared with several currently popular methods on 18 external human datasets and 29 external mouse datasets, and the experimental results show that scTransSort outperforms other methods with its high accuracy and high performance. scTransSort represents the gene expression matrix as a gene expression-embedding block and uses an improved transformer model to extract effective features from the data for cell type identification. This method is efficient, accurate, and does not require additional references beyond the scRNA-seq data. scTransSort provides more possibilities and expandable directions for the analysis of single-cell data.

## 2. Materials and Methods

### 2.1. Datasets

To explore the scalability of the model, experiments were conducted using a large number of scRNA-seq datasets from different species, different tissues, and different platforms. All scRNA sequence datasets used in this paper are published and publicly available datasets from multiple high-quality reports and the Gene Expression Omnibus (GEO). Used as a human cell dataset are 295,805 cells from 35 tissues of the human cell landscape (HCL, https://figshare.com/articles/dataset/HCL_DGE_Data/7235471, accessed on 1 June 2022) and as a mouse cell dataset are 105,148 cells from 26 tissues of the mouse cell atlas (MCA, https://figshare.com/articles/dataset/MCA_DGE_Data/5435866, accessed on 1 June 2022), with 103,148 cells from 26 tissues [33]. Statistical information on the dataset, including the number of tissues, cells, genes, and cell types, can be obtained from Supplementary Table S1. External test sets for comparing scTransSort with other methods are freely available from the Public platform, as detailed in Supplementary Table S2. We performed experiments using the data processed by Shao X et al. [33], in which the gene names of humans and mice were represented according to NCBI gene data (https://www.ncbi.nlm.nih.gov/gene/, accessed on 1 June 2022) uniformly and mismatched, and duplicated genes were removed. The data are normalized using the “LogNormalize” method by globally scaling the gene expression values for each cell by the total expression values, multiplying them by a scaling factor (default 10,000), and finally, log-transforming the results.

### 2.2. Framework of scTransSort

The high dimensionality and sparsity of scRNA-seq data are currently the main challenges in performing a single-cell data analysis. In this paper, based on the similarities between gene expression profiles and visual image systems, we propose a method for representing genes as gene-embedding blocks, which reduces the sparsity of scRNA-seq data. The scTransSort is built by combining gene embedding with a deep learning method of the transformer. Unlike traditional input sequences, genes do not have an inherent order. The transformer architecture based on the self-attention mechanism is able to learn the relationships between sequence elements efficiently, capable of handling unordered inputs, focusing on complete sequences, and learning long-term relationships. This approach brings significant advantages for unordered large-scale datasets such as gene transcriptome data [39].

**Figure 1:**
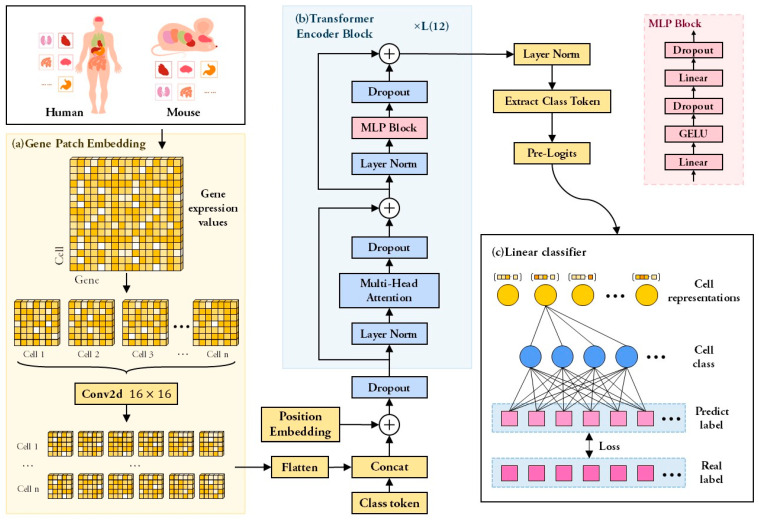
The architecture of the scTranSort. (**a**) The gene patch-embedding layer. Receive scRNA-seq data, transform it into a two-dimensional matrix of gene expression, and generate gene-embedding patches; (**b**) The transformer encoder block. It consists of a multi-head self-attention mechanism and a fully connected feedforward network to obtain a high-dimensional vector representation of the input sequence; (**c**) The linear classifier layer. The input represented by the high-dimensional vector is mapped to a set of cate-gory probabilities to obtain the final classification result.

The framework of scTransSort is shown in Figure 1. The scTransSort model mainly consists of a gene patch embedding, a transformer encoder, and a linear classifier. First, at the gene patch-embedding layer, scTransSort receives the scRNA-seq data and stores the data for each cell as a two-dimensional matrix of gene expression. By using CNN to generate gene-embedding patches, we reshape the scRNA-seq data $x\in R^{C\times G}$ (C is the total number of cells, and G is the total number of gene expression) into a sequence of flattened 2D patches $x_{P}\in R^{N\times (P^{2})}$(P is the edge size of each patch, and N=C×GP2 is the number of patches obtained). Position embedding is added to each patch to fix the relative position relationship in space between genes and genes to extract global features of gene expression data using the possible structural relationships of scRNA-seq data. Inputting scRNA-seq data into the transformer model in the form of gene-embedding blocks can effectively avoid the problems of high complexity and high training computational resources caused by the long input sequences. The high-level features of the gene expression cell types are then extracted and propagated to the linear classifier using 12 layers of transformer encoder blocks, and the prediction confidence of each cell type is ultimately output. The transformer encoder is composed of layers that alternate between multi-head attention and multilayer perception (MLP) before each block, LayerNorm (LN) is applied, and the residual connections are applied after each block Vaswani et al., 2017 [40]; Wang et al., 2019 [41]; Baevski & Auli 2019 [42].

The linear classifier layer consists of a three-layer MLP, which is composed of multiple linear layers and nonlinear activations [43]. The high-level features output by the transformer encoder block are used as input to the MLP and, by assigning a confidence level to each cell type, the final output of the predicted cell type results. The gaussian error linear units (GELU) are used as the activation function. Since the cumulative distribution function of GELU is usually calculated using the error function, GELU is defined as

(1) $$GELU\left(x\right)=xP\left(X\leq x\right)=x\Phi \left(x\right)=x\int_{-\infty }^{x}\frac{e^{-\frac{{\left(X-\mu \right)}^{2}}{2\sigma^{2}}}}{\sqrt{2\pi }\sigma }dX$$

where x is the current neuron’s activation value input, $and\;\Phi \left(x\right)$ is the cumulative distribution of the gaussian normal distribution of x.

### 2.3. Loss Function and Parameters Setting

The SparseCategoricalCrossentropy function is used as the loss function in our model, which is defined as:

(2) $$L=-\frac{1}{n}\sum_{i}^{n}\left(y_{i}log{\hat{y}}_{i}+(1-y_{i})log(1-{\hat{y}}_{i})\right)$$

where vector $y_{i}$ is the true label for the ith cell, vector ${\hat{y}}_{i}$ is the predicted label for the ith cell, i is the cell sample, and n is the total number of cell samples.

Table 1 describes the parameter settings of the model. TensorFlow 2.4.0 is used to implement the neural network model, and Python 3.6 is used to write the code. Seed is used to initialize the network’s parameters. The network is trained over 50 epochs using a batch size of 64 samples, which is the number of samples used in each global step. Using the cosine learning rate decline approach, the decay strategy of the learning rate is customized, and it can be expressed as follows:

(3) $$r_{t}=0.5\times r_{0}\left(1+cos\left(\frac{t\pi }{T}\right)\right)$$

where $r_{0}$ is the initial learning rate, t is the current step number, and T is the number of steps after which the learning rate decays to 0.

## 3. Results

### 3.1. Evaluation Metrics

To demonstrate the advantages and scalability of scTransSort, we evaluate the performance of the model using different evaluation metrics, including precision, recall, Accuracy (ACC), Matthews correlation coefficient (MCC), and F_1-score_. Since we are solving a multi-category problem with unbalanced data in each category, we choose macro precision, macro recall, and macro F_1-score_. These metrics have different emphases. Accuracy indicates the percentage of correct prediction types across all cells and focuses on assessing the ability of the model to correctly classify samples. In contrast, the macro F_1-score_ focuses on assessing the sensitivity of the model. The MCC focuses on predicting the classification performance of models in unbalanced datasets. A dataset containing at least two cell types was selected to calculate the macro F_1-score_ and MCC. All the evaluation metrics used are detailed in Table 2. The evaluation parameters used in this paper, TP, FP, FN, and TN, represent positive samples predicted by the model to be positive, negative samples predicted by the model to be positive, positive samples predicted by the model to be negative, and negative samples predicted by the model to be negative, respectively.

### 3.2. Performance on Internal Datasets

**Figure 2:**
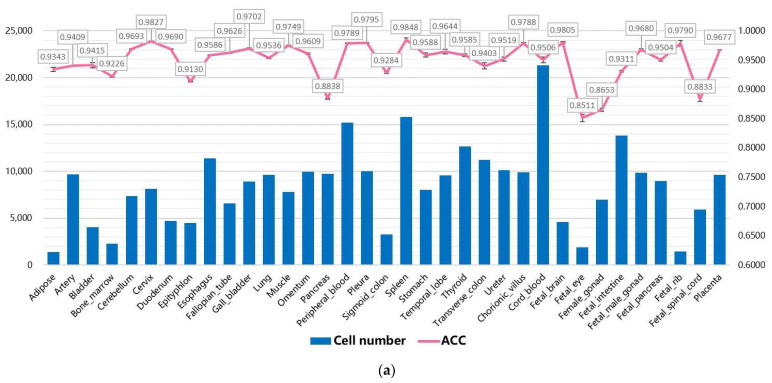

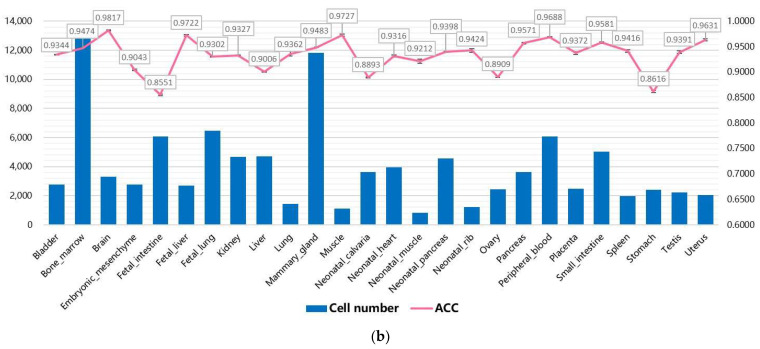
Performance of scTransSort on internal test datasets. (**a**) Accuracy of scTransSort in annotating cells from 35 human tissues. (**b**) Accuracy of scTransSort in annotating cells from 26 mouse tissues. The bar graph shows the number of cells per tissue.

To evaluate the classification performance of scTransSort on cells from different tissues, we conducted experiments on 295,805 cells from 35 human tissues and 103,148 cells from 26 mouse tissues, respectively. The dataset is randomly divided into training and test sets according to the ratio of 8:2, and each random split was performed for 5 replicate experiments. The training set is used to train the model. The test set is for testing the generalization ability of the model. The experimental results are shown in Figure 2.

It can be seen that scTransSort performs very well on both human and mouse tissues. The average accuracy of scTransSort ranged from 85.11% to 98.48% on 35 datasets from human tissues and 85.52% to 98.17% on 26 datasets from mouse tissues. Even on small datasets with a relatively small number of cells, such as Adipose, Bone_marrow, Fetal_rib, Lung, Muscle, Spleen, and Uterus, the average accuracy of scTransSort predicting cell types is above 90%. Another aspect can be found that scTransSort performs similarly on most of the same tissue datasets from two different species, such as bladder, lungs, muscles, spleen, uterus, etc., in humans and mice. Experimental results on these different tissues demonstrate the high performance and broad applicability of scTransSort for cell-type identification tasks.

**Figure 3:**
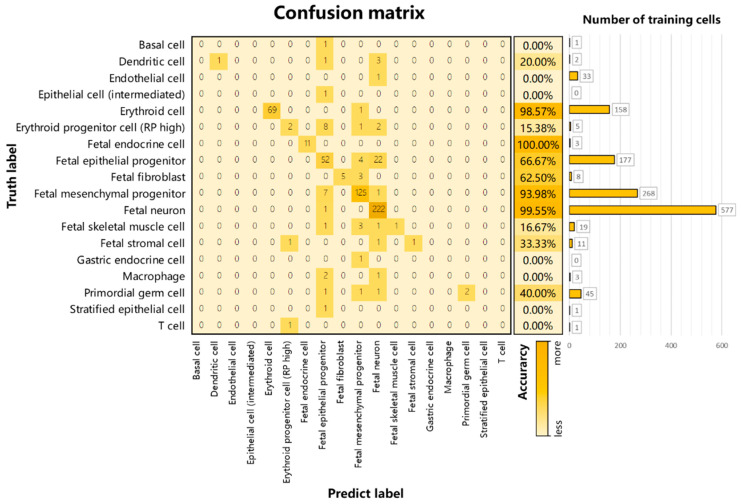
Confusion matrix of scTransSort classification results on the human fetal eye dataset. The accuracies in the graphs represent the accuracy of the predicted results for each cell label, respectively. The bars represent the number of cells in the training dataset for each cell label.

Although scTransSort showed a strong classification performance in most human and mouse tissues, the performance was less than perfect in individual tissues, such as human fetal eye, human female gonad, and mouse fetal intestine. We construct the confusion matrix of the classification results using fetal eye tissue with an accuracy of 85.11% in Figure 2a as an example. The confusion matrix clearly shows exactly which part of the classification model was confused when performing the classification prediction task, providing insight into the errors made by the model and overcoming the limitations of using classification accuracy alone to analyze prediction results. As shown in Figure 3, it can be clearly seen that the labels with the highest classification accuracy are fetal endocrine cell, erythroid cell, fetal neuron, and fetal mesenchymal progenitor. By comparison, it is easy to see that cell labels trained with more cell samples have relatively high accuracy, while those with very low accuracy usually have only few training cell samples. On the other hand, the model tends to predict cells with unclear labels as cellular labels with larger training sets, such as fetal epithelial protoplasts. The cell classification results for fetal epithelial progenitor were only 66.67% accurate, with 28.21% of cells misclassified as fetal neurons with the highest number of trained cells and 5.13% of cells misclassified as fetal mesenchymal progenitors with the second-highest number. By these circumstances, it can be speculated that the reason for the relatively poor performance of scTransSort on individual tissues such as human fetal eye tissue may be due to the insufficient number of training samples, the model does not extract features comprehensively enough on some cell labels and does not learn sufficiently.

### 3.3. Performance and Robustness Compared with Other Methods

A comprehensive comparison of scTransSort’s performance with that of scDeepSort, singleCellNet, ACTINN, CHETAH, SVM, scPred, SCINA, scMap_cell, SingleR, scID, CellAssign, scMap_cluster, and Garnett is presented. Specifically, to compare the generalization ability of the models, we pretrained the models on an internal training set and then performed experiments on an external test set of 117,940 cells from nine human tissues and 67,617 cells from 12 mouse tissues for cell type annotation, respectively. The detailed correspondence of the datasets can be found in Supplementary Table S3.

**Figure 4:**
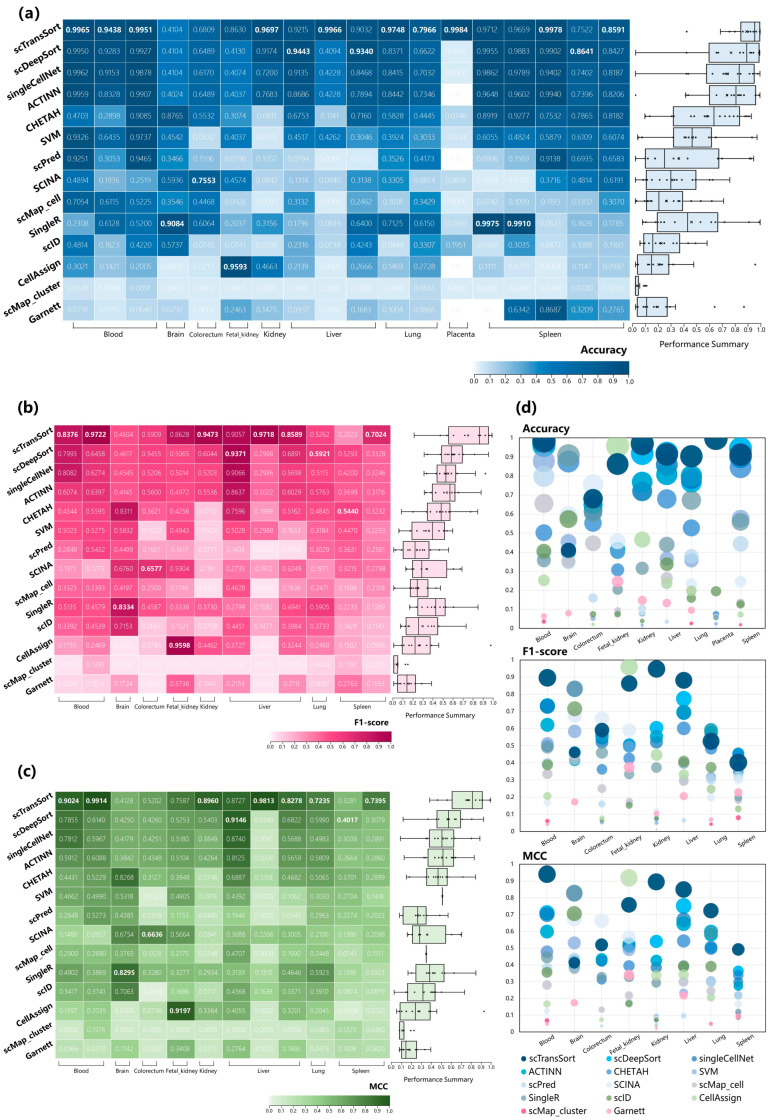
Performance comparison of scTransSort on human external test datasets (accessed on 1 June 2022). (**a**) Heat maps and boxplots of accuracy comparison for different methods on 18 datasets from 9 tissues; (**b**) Heat maps and boxplots of the mean F1 score comparison; (**c**) Heat maps and boxplots of the mean MCC comparison. The bolded font indicates the top-ranked method for each dataset; (**d**) The bubble charts summarize the accuracy, mean F1 score, and mean MCC of the different methods in each tissue.

**Figure 5:**
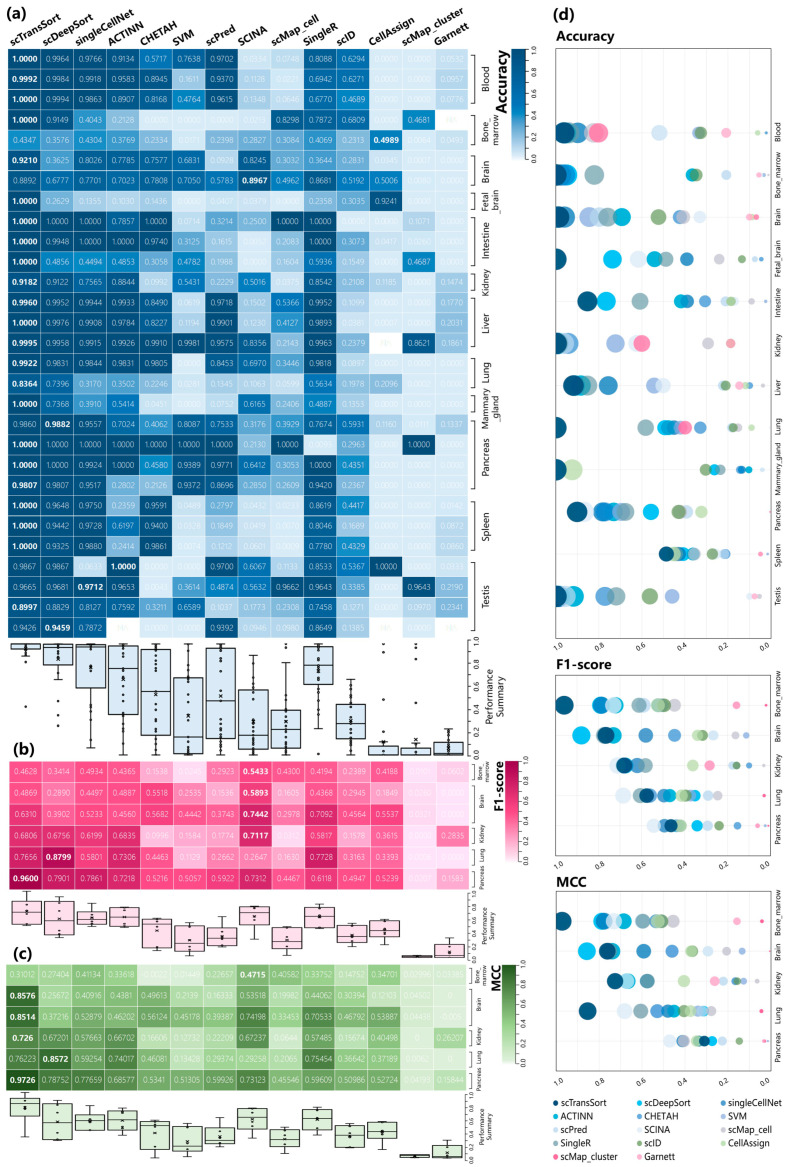
Performance comparison of scTransSort on mouse external test datasets (accessed on 1 June 2022). (**a**) Heat maps and boxplots of accuracy comparisons for different methods on 29 datasets from 12 tissues; (**b**) Heat maps and boxplots of the mean F1 score comparison; (**c**) Heat maps and boxplots of the mean MCC comparison. The bolded font indicates the top-ranked method for each dataset; (**d**) The bubble charts summarize the accuracy, mean F1 score, and mean MCC of the different methods in each tissue.

The experimental results on human tissues and mouse tissues are shown in Figure 4, and Figure 5, respectively, and part of the experimental results are taken from the references of Shao X. et al. [33]. Each set of experiments was repeated five times, and the average of the results of each five experiments is shown in the figure. It can be seen that scTransSort outperforms other methods overall for each tissue in the 18 human and 29 mouse datasets. Specifically, the scTransSort method achieves the highest accuracy on most of the datasets, and besides, the F1 average score and MCC average are also successful. In addition, as seen in the bubble chart, scTransSort outperforms other methods overall, both in human and mouse tissues. It can be found that CellAssign and Garnett perform poorly, which may be due to the heavy dependence on the quality and integrity of the selected marker genes. Although the comprehensive performance of scID, CellAssign, scMap, and Garnett on all datasets is much worse than other methods, they have shown good performance on some datasets, such as the spleen in the human dataset and testis in the mouse dataset.

**Figure 6:**
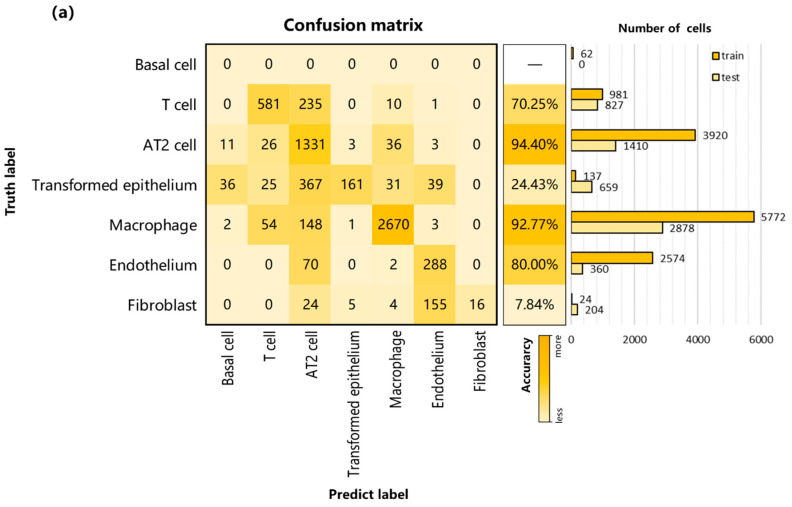

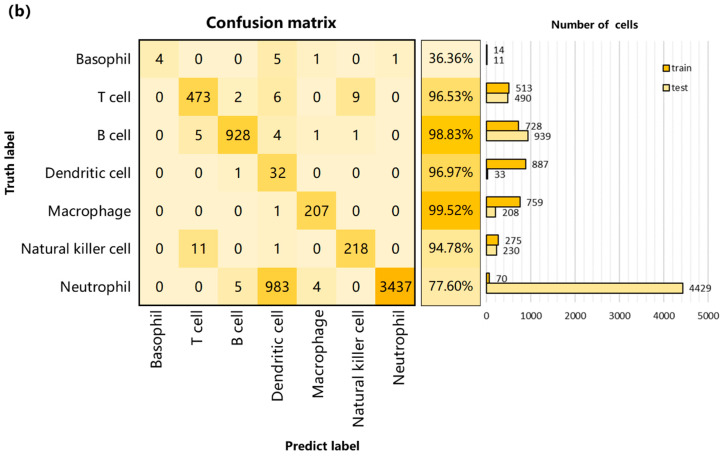
Confusion matrix of prediction results on external test datasets. (**a**) Confusion matrix for predicting results on the human lung dataset; (**b**) Confusion matrix for predicting results on the mouse lung dataset. The accuracies in the graphs represent the accuracies of the predicted results for each cell label. The bars represent the number of training cell samples and the number of test cell samples for each cell type.

It is evident that scTranSort has the best overall performance, but it can also be observed that, in some specific datasets, the predicted results are not optimal. To analyze this, we take human and mouse lung tissues as examples and construct a confusion matrix on an external test set to visualize the classification results. As shown in Figure 6a, in human lung tissues, the AT2 cells, macrophage, and endothelium have good classification results. However, the predicted results for transformed epithelium and fibroblast are not satisfactory. By observation, it can be concluded that the reason for this is due to the low number of cell samples in the training sets for these categories. Specifically, the training set for transformed epithelium only has 137 cell samples, while the test set has 659 cell samples, resulting in an accuracy of only 24.43%. Similarly, fibroblasts only have 24 cell samples in the training set, resulting in inadequate training and very low accuracy. Similar conclusions can be drawn for mouse lung tissues, as shown in Figure 6b. Several categories, such as macrophages, B cells, dendritic cells, T cells, and natural killer cells, have good prediction results, as they have relatively sufficient training set sizes. However, basophil and neutrophil cell types have low training set sizes, with only 14 and 70 samples, respectively, resulting in poor classification results. When the number of samples in certain categories in the training set is small, the model may only learn local patterns in these categories’ data, which can lead to poor performances on the test sets of these categories and a failure to capture the overall data distribution features. To improve the performance and reliability of the model, we will collect and organize training set data containing a more comprehensive range of cell types in the future to address the issue of imbalanced data.

### 3.4. The Effect of Feature Order of Input Data on Model Performance

**Figure 7:**
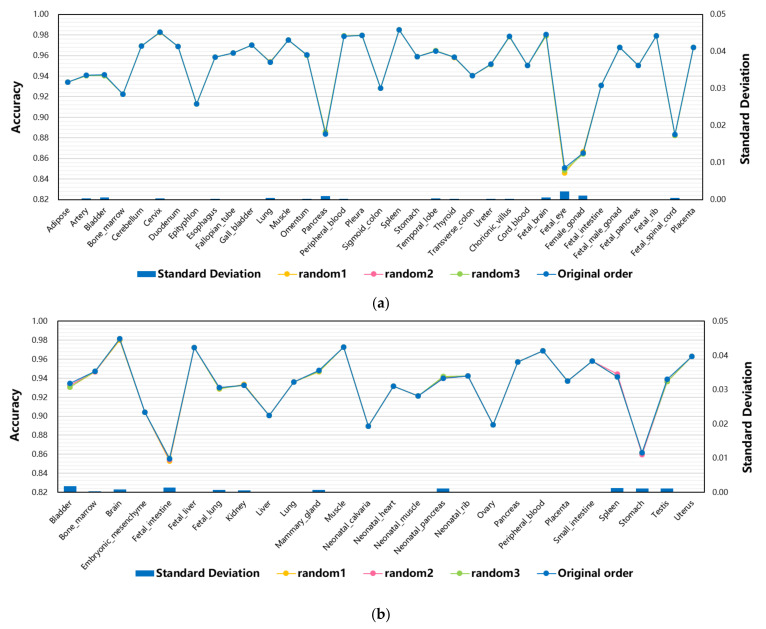
Performance of scTransSort under different inputs. (**a**) Experiments on cells containing 35 human tissues; (**b**) Experiments on cells containing 26 mouse tissues. The line chart shows the accuracy of cell classification predictions for each group of data under different input modes. Each color line represents a specific input mode. The bar chart displays the standard deviation of the results obtained with different inputs.

By randomly disordering the sequencing data for each cell, we compared the predicted labels given by scTransSort on four different input patterns (different random disordering). Each experiment was repeated three times on separate internal datasets, consisting of 35 human tissues and 26 mouse tissues. The experimental results are shown in Figure 7, and it can be seen that the accuracy of the scTransSort prediction results is not significantly different on different input data patterns, whether on the dataset of human or mouse tissues. As can be seen, scTransSort’s predictive power is not affected by the internal order of the input data. scTransSort intelligently grabs information from disordered data, extracts effective features autonomously, and makes accurate predictions.

### 3.5. The Effect of Different Patches on Model Performance

**Figure 8:**
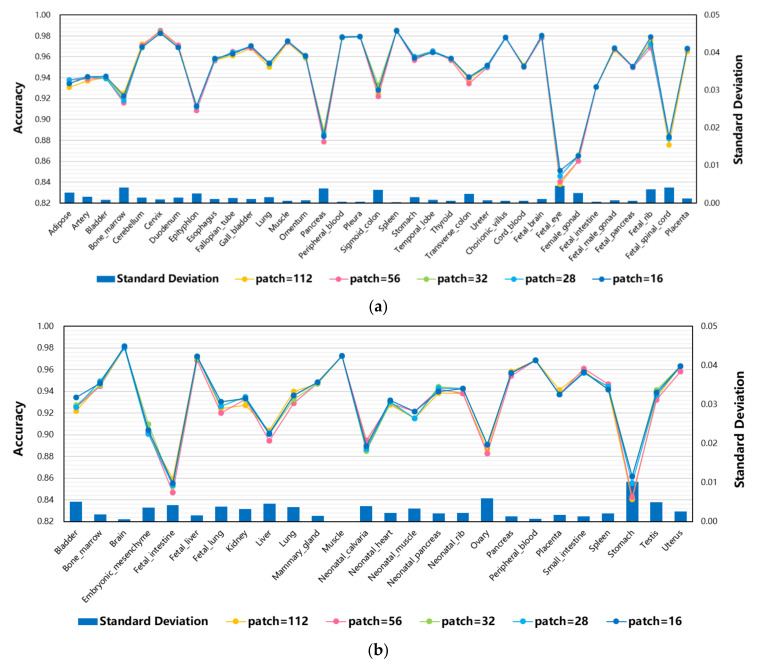
Performance of scTransSort at different patch parameter settings. (**a**) Experiments on cells containing 35 human tissues; (**b**) Experiments on cells containing 26 mouse tissues. The line chart shows the accuracy of cell classification predictions for each group of data under different model parameter settings (patch size). Each color line represents a specific parameter setting. The bar chart displays the standard deviation of the results obtained with different patch parameter settings.

It is necessary to consider the impact of different patch sizes on the model. A number of gene-embedding patch sizes were tested on the performance of scTransSort for cell type annotation. As shown in Figure 8 of the experimental results, differences in performances between human and mouse tissues are not significant, indicating that the batch size does not affect the performance significantly. The experimental results illustrate the strong robustness of scTransSort. However, smaller gene-embedding patches will result in a better performance, as large batches will have a higher risk of data sparsity when constructing gene embeddings. In other experiments, the size of the patch is set to 16 by default.

## 4. Discussion

In this study, we propose a deep neural network model scTransSort based on the transformer idea of fused gene embedding for cell type annotation, which does not require manually labeled features and can intelligently grab information from unordered data using a multi-head attention mechanism to learn effective features from the data autonomously with strong robustness and high accuracy. Our method is more effective than other methods in performing cell classification tasks on external datasets of 18 humans and 29 mouse, as evidenced by higher accuracy, high robustness, and better generalization.

Furthermore, through extensive experiments on a total of 80 independent datasets from 35 human and 26 mouse tissues, respectively, it can be shown that scTransSort can accurately and efficiently predict most cells in human and mouse tissues, showing a high performance on the cell type classification task. In summary, scTransSort has the outstanding ability to learn complex relationships in unordered data to extract valid features and has achieved high accuracy in numerous prediction tasks, which also demonstrates the feasibility of the transformer idea in single-cell classification tasks. scTransSort has great potential for the task of identifying cell types.

Although scTransSort has demonstrated its excellent performance, it can be noted that the prediction accuracy is still limited on some datasets, and it is hoped that this problem can be solved by collecting more training data in the future. In addition, the biological interpretation of the transformer model should be improved in the future, and further efforts should be made to the interpretability of the model, which will greatly facilitate the development of biological research and biomedical applications.

Despite the outstanding performance of scTransSort, it is worth noting that the prediction accuracy is still limited in certain datasets. Collecting more training data may help address this issue in the future. Additionally, there is a need to enhance the biological interpretability of the transformer model, which could greatly promote biological research and biomedical applications.

On the other hand, our experiments have shown that scTransSort performs exceptionally well in processing sequence data such as scRNA-seq, which not only contains information on cell types but also on cell states and cell cycles. As such, it is possible that a transformer-based model can learn the expression patterns of cells at different time points with high predictive power. Our next step is to collect and organize a high quality dataset that includes cell cycle stages and cell states and explore the algorithm’s effectiveness in identifying cell cycles and discriminating cell states. We anticipate that future research in this area will provide even more valuable information for cell biology and medical research.

## Figures and Tables

**Table 1:** Parameter values in scTransSort.

Parameters	Range
patch_size	16
batch_size	64
epoch	50
initial_lr	1 × 10^−3^
end_lr	1 × 10^−5^
weight_decay	1 × 10^−4^
Optimizer	SGD
Activation	GeLU

**Table 2:** Evaluation parameters used in this paper.

	Actual Positive	Actual Negative
Predicted Positive	TP	FP
Predicted Negative	FN	TN
Precision	TP/(TP+FP)	
Recall	TP/(TP+FN)	
Accuracy (ACC)	(TP+TN)/(TP+FP+FN+TN)	
Matthews correlation coefficient (MCC)	MCC = $\frac{TP\times TN-FP\times FN}{\sqrt{\left(TP+FP\right)\left(TP+FN\right)\left(TN+FP\right)\left(TN+FN\right)}}$	
F_1-score_	F_1-score_ = 2$\times \frac{Precision\times Recall}{Precision+Recall}$	
Standard Deviation	s =$\sqrt{\frac{\sum_{i=1}^{n}{\left(x_{i}-\overset{-}{x}\right)}^{2}}{n-1}}$ (where n is the number of data, $x_{i}\;is$ the i-th data, and $\overset{-}{x}is\;the\;arithmetic\;mean\;of\;the\;n\;data.$)	

## Data Availability

The datasets analyzed during the current study are available at https://figshare.com/articles/dataset/HCL_DGE_Data/7235471 (accessed on 1 June 2022) (the human cell landscape) and https://figshare.com/articles/dataset/MCA_DGE_Data/5435866 (accessed on 1 June 2022) (the mouse cell atlas). The code underlying the article is available at https://github.com/jiaojiao-123/scTransSort (accessed on 1 December2022).

## Supplementary Materials

The following supporting information can be downloaded at: https://www.mdpi.com/article/10.3390/biom13040611/s1, Table S1: Statistical information for the internal training data set, including species, tissue, cell count, gene count, and cell type count, Table S2: Cell type mapping relationships between human and mouse training data and external test data, including pedigree dependent and marker dependent approaches, Table S3: Statistical information on training data and external test data sets for humans and mice, including species, tissue, cell count, gene count, cell type count, PMID, and public data source or platform.
