# Supplementary material for: scTransSort: Transformers for Intelligent Annotation of Cell Types by Gene Embeddings
Source: Biomolecules. 2023 Mar 28;13(4):611. doi: 10.3390/biom13040611 (PMC10136153; doi:10.3390/biom13040611)
Supplement: Supplementary file 1 [file biomolecules-13-00611-s001.zip › Supplementary Table legends.docx]

**Supplementary Table S1.** Statistical information for the internal training data set, including species, tissue, cell count, gene count, and cell type count.

**Supplementary Table S2.** Cell type mapping relationships between human and mouse training data and external test data, including pedigree dependent and marker dependent approaches.

**Supplementary Table S3.** Statistical information on training data and external test data sets for humans and mice, including species, tissue, cell count, gene count, cell type count, PMID, and public data source or platform.
